# Molecular analysis in a GALNS study cohort of 15 Tunisian patients: description of a novel mutation

**DOI:** 10.1186/s13000-016-0498-y

**Published:** 2016-06-17

**Authors:** Latifa Chkioua, Souhir Khedhiri, Hind Hafsi, Oussama Grissa, Hadhami Ben Turkia, Abdelhedi Miled, Sandrine Laradi, Roseline Froissart, Najat Alif

**Affiliations:** Laboratory of Biochemistry, F. Hached Hospital, 4000 Sousse, Tunisia; University of Monastir, 5000 Monastir, Tunisia; Targeted Therapy Team, The Institute of Cancer Research, London, United Kingdom; Laboratory of pediatrics, La Rabta Hospital, 1007 Tunis, Tunisia; The Auvergne-Loire Regional Branch of the French National Blood System EFS/GIMAP-EA 3064, 42100 Saint Etienne, France; Hereditary Metabolic Diseases Service. Center for biology and pathology, Est Hospices Civils, Lyon, 69677 Bron cedex, France; Department of Biology, Laboratory of Biotechnologies and Valorization of Natural Resources. IBN Zohr University, School of Sciences, BP 8106 Agadir, Morocco

**Keywords:** Mucopolysaccharidosis type IVA, *N*-acetylgalactosamine-6-sulfatase, Mutation screening, Bioinformatics analysis, Haplotypes

## Abstract

**Background:**

Mucopolysaccharidosis type IVA (MPS IVA) is an autosomal recessive disease caused by the deficiency of the lysosomal enzyme *N*-acetylgalactosamine-6-sulfate sulfatase (*GALNS).* The purpose of this study was to analyze the *GALNS* mutations and the haplotypes associated.

**Methods:**

Mutation screening of the *GALNS* gene was performed by direct sequence analysis using DNA samples from 15 unrelated Tunisian MPS IVA patients. We also analyzed the haplotypes associated with the novel mutation and with the other reported *GALNS* mutations.

**Results:**

We have identified an unreported missense mutation p.D288G (c.863A > G) in one patient, the most frequently c.120 + 1G > A (IVS1 + 1G > A) mutation in eleven MPS IVA patients and three previously reported mutations p.G66R, p.A85T and p.R386C on the other MPS IVA patients. All the studied patients were homozygous for these identified mutations. Bioinformatics analysis predicted the novel mutation as being probably pathogenic. These findings with the unobserved p.D288G mutation in controls subjects, suggested that it is a disease-causing mutation, which was correlated with the severe phenotype observed in the patients. We have found that the two *GALNS* unreported and reported mutations, respectively p.D288G and p.R386C, were associated with a common and specific haplotype.

**Conclusion:**

Our results were in agreement with previous reports from Tunisia, suggesting, on one hand the genotype/phenotype correlations in MPS IVA patients and the other hand the haplotype analyses were useful for determination of mutation origin in Tunisian population.

## Abstract (fr)

**Résumé:**

La mucopolysccharidose de type IV A (MPS IVA) est une maladie autosomique récessive due à la déficience de l’enzyme lysosomale *N*-acétylgalactosamine-6-sulfate sulfatase *(GALNS*). Le but de cette étude est d’analyser les mutations du gène *GALNS* et les haplotypes associés.

**Méthodes:**

Le dépistage des mutations dans le gène GALNS a été réalisé par l’analyse directe des séquences en utilisant des échantillons d’ADN issus de 15 patients non apparentés atteints de la MPS IVA. Par ailleurs, nous avons analysé les haplotypes associés, d’une part à la mutation nouvelle, et d’autre part, aux autres mutations déjà rapportées.

**Résultats:**

Nous avons identifié une nouvelle mutation faux-sens p.D288G (c.863A > G) chez un patient, la mutation la plus fréquente c.120 + 1G > A chez onze patients et trois mutations déjà rapportées : p.G66R, p.A85T et p.R386C chez trois patients. Tous les patients étudiés sont homozygotes pour ces mutations identifiées. Les analyses bioinformatiques prédisent que la mutation, p.D288G, décrite pour la première fois pourrait être pathogénique. L’absence de cette nouvelle mutation chez la population témoin suggère que la mutation p.D288G, corrélée au phénotype sévère chez le patient étudié, est responsable de la maladie. Par ailleurs, nous avons constaté que les deux mutations du gène *GALNS*, nouvelle et déjà rapportée, respectivement, p.D288G et p.R386C sont associées à un haplotype commun et spécifique.

**Conclusion:**

Nos résultats sont en accord avec les rapports précédents de la Tunisie, ce qui suggère, d’une part les corrélations génotype/phénotype chez les patients MPS IVA et d’autre part l’analyse des haplotypes ont été utiles pour la détermination de l’origine de la mutation dans la population tunisienne.

## Background

Mucopolysaccharidosis type IVA (MPS IVA) or Morquio A disease (MPS IVA; OMIM 253000) is a metabolic lysosmal and autosomal recessive disease resulting from the deficiency of the enzyme *N*-acetylgalactosamine-6-sulfate-sulfatase (*GALNS*: E.C.3.1.6.4), and the accumulation of undegraded or partially degraded glycosaminoglycans (GAGs): keratan sulfate (KS) and chondroitin-6-sulfate (C6S) in lysosme. Phenotypes vary from the classical form (life span of 20 to 30 years old) with corneal opacity, short trunk dwarfism, heart valvular disease, and severe bone dysplasia to attenuated forms with normal life span, mild bone and visceral complications. There is no impairment of the central nervous system in all Morquio A phenotypes [1, 2].

The *GALNS* gene, located on chromosome 16q24.3, contains 14 exons spanning 50 kb (GDB accession ID: 129085) and is transcribed into a 1566 bp cDNA, which encodes a 522-residue glycopeptide.

To date, more than 200 different mutations associated with MPS IVA have been identified [Human Gene Mutation Database (HGMD, 2014); http://www.hgmd.org/] including point mutations, splice-site mutations, deletions and insertions. This heterogeneity may explain the important phenotypes variability observed in MPSIVA patients [2–4].

Consanguineous marriages are associated with a higher frequency of autosomal recessive disorders. Thus, firstly an association between parental consanguinity and the frequency of MPS IVA exists [2], secondly the impact of consanguinity in MPS IVA was treated in several studies highlighting the major role of intermarriage in the recurrence to have haplotype [2]. The incidence of MPS IVA in Tunisia is also high, estimated at 0.45 in 100.000 live births [5].

In the present paper, we report the molecular analysis of the *GALNS* gene in 15 MPS IVA Tunisian patients using genomic DNA samples. We identified a novel unreported mutation in addition to the previously reported ones. All the genetic lesions correlated with specific haplotype contributing to a better understanding of the founder effect of these mutations.

## Methods

### Patients

The study was carried out in 15 MPS IVA patients recruited from pediatric departments of different hospitals in Sousse, Sfax and Sidi Bouzid. All studied patients were from a consanguineous marriage, and there were no relationship known between the families. All procedures were in accordance with the ethical standards of the responsible committee on human experimentation (institutional and national) and with the Helsinki Declaration of 1975, as revised in 2000 and approved by the Ethics Committees of the respective Tunisian hospitals. Informed consent was obtained from all patients and their families before being included in the study. Additional informed consent was obtained from all patients for whom identifying information is included in this article.

### Biochemical assay

The diagnosis of the MPSIVA disease was based on the following approach after a clinical and paraclinical suspicion.

#### Quantitative analysis of total urinary glycosaminoglycans

We first performed the study of urinary GAGs. Urinary GAGs were quantified using a dimethylmethylene blue (DMB) test [6].

Electrophoresis on cellulose acetate plate was performed to identify the types of GAGs present in excess (*e.g.*, dermatan sulfate, heparan sulfate, keratan sulfate). Discontinuous electrophoresis on cellulose acetate plates separated the different GAGs based on their charge and differential solubility in ethanol. The mucopolysaccharides were visualized by staining with alcian blue [6].

#### GALNS enzyme assay

The *GALNS* enzymatic activity was measured in sonicated fresh leukocyte pellets using the fluorogenic substrate 4-methylumbelliferyl-β-D-galactoside-6-sulfate, as previously described [7].

### Molecular analysis

#### GALNS mutation analysis

Genomic DNA was isolated from venous blood by the phenol/chloroform procedure, according to standard protocols as previously described [8].. Genomic DNA of *GALNS* gene was amplified and sequenced. For patients with a family history of known or predicted pathogenic mutations or for the index cases’ parents, targeted PCR and sequencing were performed in the Laboratory of Inherited Metabolic Diseases in Lyon-France.

#### Haplotype analysis

In order to estimate the origins of the novel missense mutation p.D288G and the other known mutations, haplotypes were determined using *GALNS* Sanger sequencing of six amplified genomic fragments, each one including one polymorphism [9, 10].

The mutant and normal alleles were tested for six polymorphisms. These included the following known polymorphisms in: (1) intron 5 (IVS5 + 134; CCAAGG [allele A] or CCGAGG [allele a] [11], (2) exon 7 (763 nt from A of the ATG initial codon on the cDNA; GCACGC [allele B] or GCATGC [allele b] [12], (3) intron 7 (IVS7nt90; GTAC [allele H] or GAAC [allele h] [11], (4) exon 11 at cDNA 1232 (GTCC [allele C] or GGCC [allele c]) [11], (5) exon 13 at cDNA 1487 (AAGCCT [allele D] or AGGCCT [allele d] [11], and (6) exon 14 (CCAG [allele E] or CCGG [allele e] [11].

## Results

The clinical features of each patient are presented in Table 1.

**Table 1:** Molecular and clinical findings of the fifteen MPS IVA patients

	Cases														
	1	2	3	4	5	6	7	8	9	10	11	12	13	14	15
Sex	Male	Female	Male	Female	Female	Female	Female	Male	Female	Male	Female	Male	Female	Female	Male
Age at diagnosis (year/month)	4/5	3	8	3	5/4	7/7	3	4/4	5	3	8	5/4	5	4	6/8
Age of onset (year/month)	18 months	14 months	2	16 months	2/3	18 months	14 months	16 months	18 months	2	14 months	16 months	14 months	18 months	18 months
Consanguinity of the parents/degree	1st cousins	1st cousins	2nd cousins	3rd degree	2nd cousins	1st cousins	1st cousins	1st cousins	1st cousins	1st cousins	1st cousins	1st cousins	1st cousins	1st cousins	1st cousins
Symptoms	Marked	Marked	Marked	Marked	Marked	Marked	Marked	Marked	Marked	Marked	Marked	Marked	Marked	Marked	Marked
Growth retardation															
Short trunk	+	+	+	+	+	+			+			+			+
Short neck	+	+	+	+	+	+	+	+	+	+	+	+	+	+	+
Genu valgum	Marked	Marked	Marked	Marked	Marked	Marked	Marked	Marked	Marked	Marked	Marked	Marked	Marked	Marked	Marked
Corneal opacities	+	+	+	+	+	+	+	+	+	+	+	+	+	+	+
Hepatomegaly	+	-	-	+	+	-	-	+	+	+	-	-	-	-	+
*GALNS* activity nmol/h/mg leukocyte protein	0.006	0.08	0.01	0.007	0.008	0.02	0.05	0.006	0.06	0.03	0.05	0.04	0.01	0.009	0.03
Mutations	c.120 + 1G > A; IVS1 + 1G > A	c.120 + 1G > A; IVS1 + 1G > A	c.120 + 1G > A; IVS1 + 1G > A	c.120 + 1G > A; IVS1 + 1G > A	c.120 + 1G > A; IVS1 + 1G > A	c.287G > C; p.G66R	c.341C > T; p.A85T	c.1156C > T; p.R386C	c.120 + 1G > A; IVS1 + 1G > A	c.120 + 1G > A; IVS1 + 1G > A	c.120 + 1G > A; IVS1 + 1G > A	c.120 + 1G > A; IVS1 + 1G > A	c.120 + 1G > A; IVS1 + 1G > A	c.120 + 1G > A; IVS1 + 1G > A	c.863A > G; p.D288G
Haplotypes	ABcdeH	ABcdeH	ABcdeH	ABcdeH	ABcdeH	AbcDeh	AbCdEH	aBcDEH	ABcdeH	ABcdeH	ABcdeH	ABcdeH	ABcdeH	ABcdeH	aBcDEH

### Biochemical analysis

Phenotypic analysis confirmed the diagnosis of all MPS IVA studied patients. Indeed the electrophoresis on cellulose acetate plate of GAGs showed in all studied patients, the presence of keratan sulphate (KS) an abnormal band, compared to the control case, in addition to the band of chondroitin sulphate (CS).

### *GALNS* activity

Enzymatic activity was assayed in all MPS IVA patients using fluorogenic substrates revealing low or nondetectable *GALNS* activity (Table 1).

### Mutations detection

**Fig. 1:**
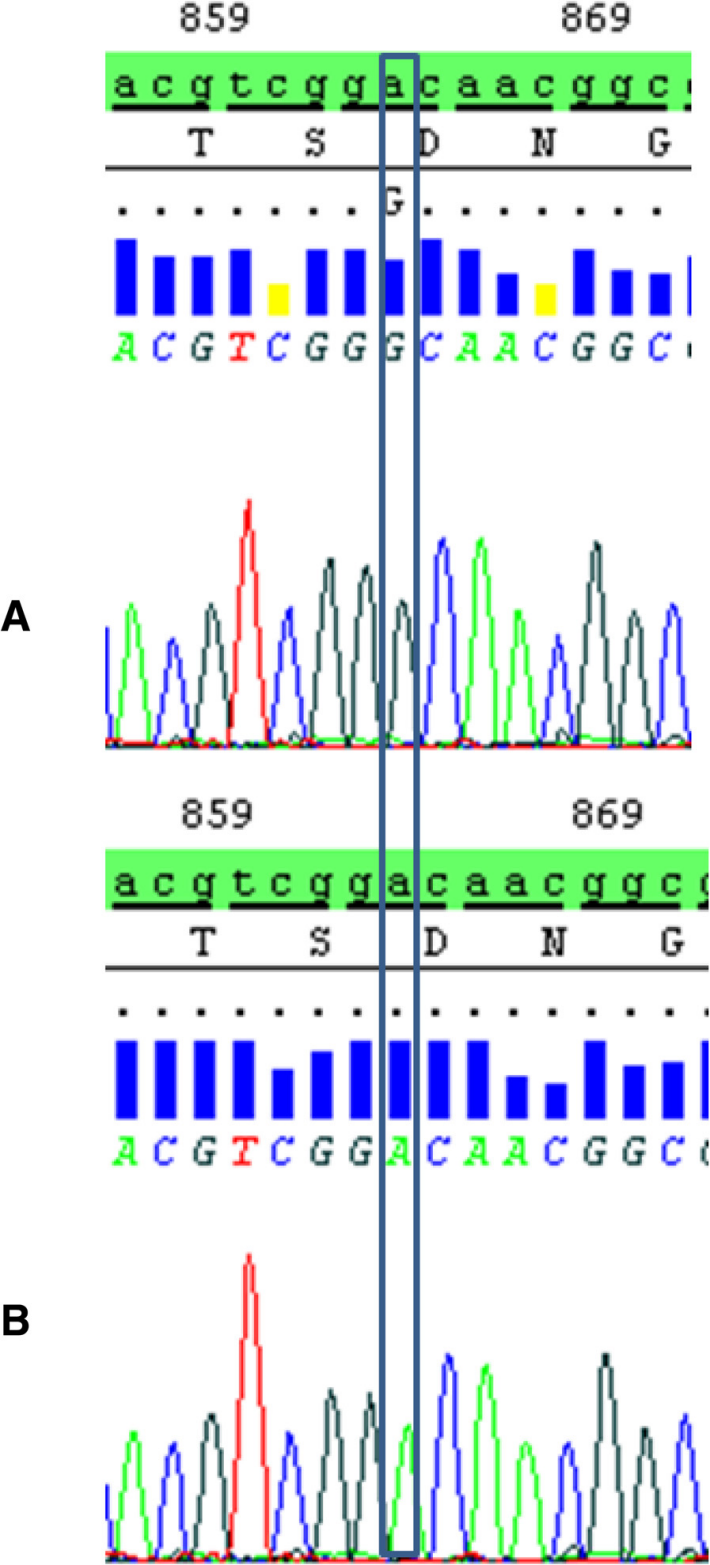
Partial nucleotide sequence of exon VIII of *GALNS* gene in MPS IVA patient homozygous for the p.D288G (**a**) and in control subject (**b**)

In this study, one patient was found homoallelic for the novel A to G transition missense mutation p.D288G (c.863A > G) (Accession#: LT576308; http://www.ebi.ac.uk/ena/data/view/LT576308), confirmed by monodirectional sequencing of the exon 8 of *GALNS* gene (Fig. 1).

**Fig. 2:**
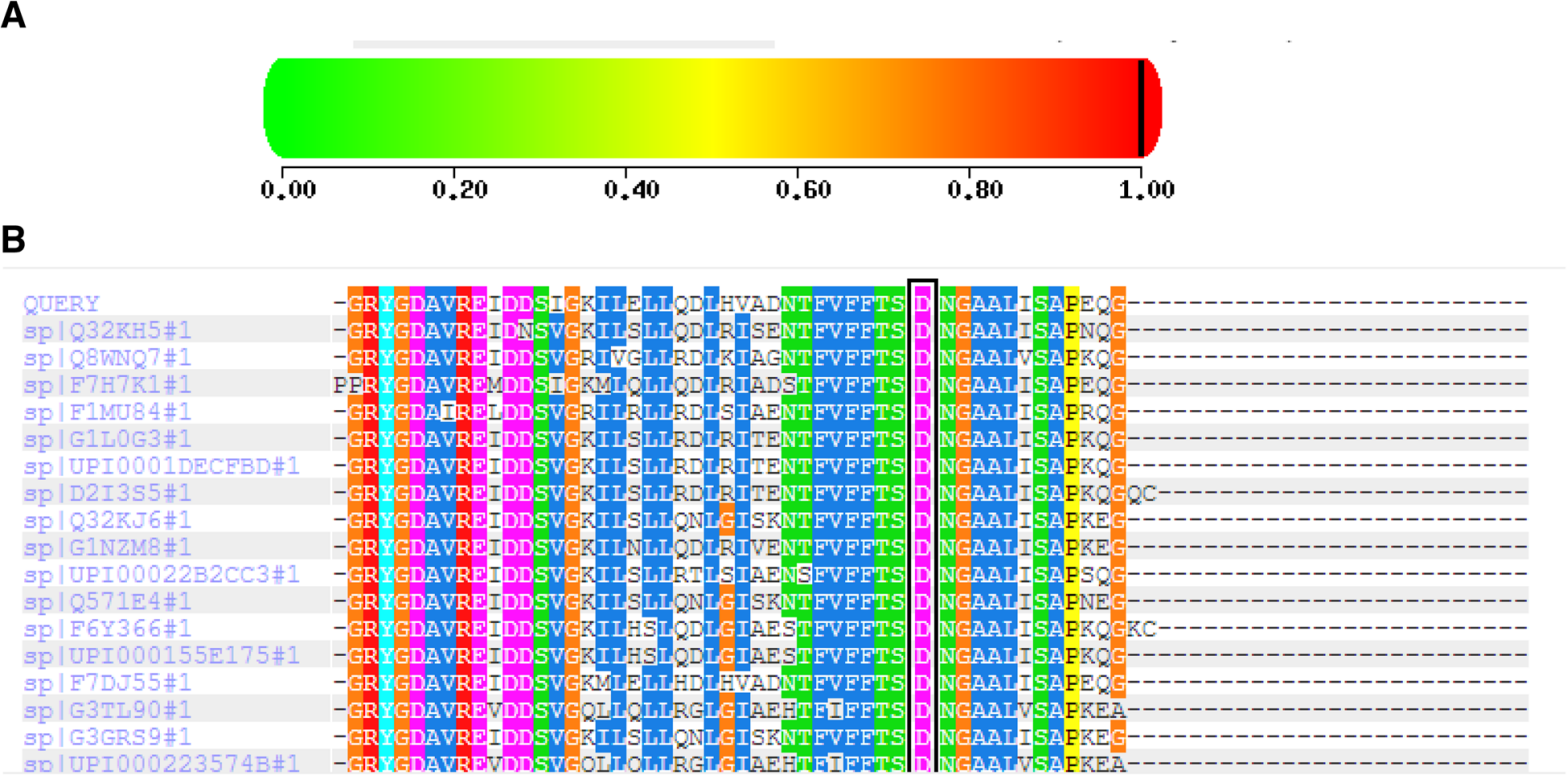
**a**: Score of damaging mutation (Polyphen v.2); **b**: multiple alignment of 17 different species (http://genetics.bwh.harvard.edu/pph2/)

The novel missense mutation p.D288G was predicted pathogenic by Polyphen v.2. The localization of the mutation was found in a conserved region among human and eukaryotic sulfatases by multiple alignments in different species (Fig. 2).

The other 14 patients were homozygous for the four previously reported mutations: c.120 + 1G > A, p.G66R, p.A85T and p.R386C. The most frequent splice site mutation c.120 + 1G > A was found in 73 % of all tested alleles. The all affected probands in this study have the typical Morquio A phenotype.

### Haplotype analysis

Haplotype analyses were performed to investigate the eventual relationships between the studied MPS IVA families with the splice site mutation and with the novel missense mutation respectively.

The control subjects (*n =* 50) presented the unique *GALNS* haplotype abcDhE. Besides, a single *GALNS* haplotype was observed (aBcDEH) in the patient (homoallelic) with the p.D288G mutation and in patients with the p.R386C mutation. In contrast, c.120 + 1G > A splice site *GALNS* mutation was always associated with a different specific haplotype ABcdeH. The p.A85T and p.G66R mutations were respectively associated with AbCdEH and AbcDeh haplotypes (Table 1).

## Discussion

In the present paper, we have screened the *GALNS* gene in 15 affected MPS IVA Tunisian patients using genomic DNA samples and performed DNA sequence analysis.

In Tunisia, the incidence for mucopolysaccharidoses type IVA is estimated at 0.45 per 100,000 live births [13]. All affected patients studied here are offspring of consanguineous marriages. First cousin inbreeding is the most represented in Tunisia [14].

In this paper, we report for the first time the occurrence of the p.D288G mutation in a homoallelic MPS IVA patient and the high incidence of the common Tunisian MPS IVA c.120 + 1G > A mutation, for which we confirmed the founder effect partially determined in a previously study [15].

The p.Asp288Gly is a non conservative mutation which leads to the substitution of an ionizable polar amino acid (Aspartate) by an apolar amino acid (Glycine) at position 288 of the GALNS protein. This mutation has been identified in a patient with a severe phenotype. It is noteworthy to precise it is located between two other previously reported mutations: p.S287L (severe phenotype [13]) and p.N289D (severe phenotype [16]). Further analysis with more number of MPSIVA patients will be important to better understand the genotype–phenotype correlation.

This mutation was not present in the 100 allele’s controls we tested, suggesting it is a disease-causing relation and could be correlated with the severe phenotype observed in the patient.

Haplotype analysis can provide a way to distinguish whether a relatively common mutation results from a founder effect or from recurrent mutations, or whether multiple haplotypes results from a founder effect, recurrent mutations or multiple haplotypes. The novel missense mutation p.D288G and the previously reported missense mutation p.R386C which was identified in Turkish patients [17] were associated with the specific homoallelic haplotype aBcDEH in two patients. The frequent abchDE haplotype initially identified within normal individuals by Tomatsu (1995) [11], was identical to the one identified in normal Tunisian controls.

In a previous study, we found that the p.W141R mutation occurred in the haplotype aBhCde and the p.L390X mutation was associated with AbhCDE [17]. These mutations were not found in Tunisian MPS IVA patients although haplotype analysis is helpful to identify the origin of different mutations.

Although the two identified mutations in this cohort were associated with two different and specific haplotypes, the relationship between the two mutations could not be established because the haplotype related to p.D288G could not be informative (found only in two patients). More MPS IVA patients with this genetic alteration must be analyzed.

The geographic distribution of studied patients shows that these MPS IVA families living in different Tunisian cities were more than 100 km away, showing the absence of relationships. However, all studied patients were from a consanguineous marriage and had the same and the specific haplotype. Therefore, there is a correlation between consanguinity and *GALNS* polymorphism suggesting that the mutant alleles were derived from a common ancestor.

The molecular results completed the biochemical exploration (electrophoresis profile and enzymatic activity) and will be very helpful in case of prenatal diagnosis which is requested due to the high inbreeding rate still encountered in Tunisia. An accurate biochemical analysis is available for the diagnosis of MPS IVA. However this test was accomplished by molecular analysis for a more comprehension of the disease-mutation relation.

## Conclusion

Our report on founder mutations provides a valuable decision making for the diagnosis and prevention of MPS IVA in Tunisia. Molecular testing was a powerful tool to investigate the role of consanguinity in all cases, confirming homozygosity in a consanguineous couple.

## Abbreviations

MPS IVA: Mucopolysaccharidosis type IVA, *GALNS: N*-acetylgalactosamine-6-sulfate sulfatase, HGMD: Human Gene Mutation Database, GAGs: Glycosaminoglycans, KS: Keratan sulfate, C6S: Chondroitin-6-sulfate, DMB: Dimethylmethylene blue.

## References

[1] Tomatsu S, Montano AM, Oikawa H, Smith M, Barrera L, Chinen Y (2011). Mucopolysaccharidosis type IVA (Morquio A disease): clinical review and current treatment. Curr Pharm Biotechnol.

[2] Khedhiri S, Chkioua L, Bouzidi H, Dandana A, Ferchichi S, Ben Turkia H, Miled A, Laradi S (2012). Mucopolysaccharidosis IVA within Tunisian patients: Confirmation of the two novel *GALNS* gene mutations. Pathol Biol.

[3] Tomatsu S, Montano AM, Lopez P, Trandafirescu G, Gutierrez MA, Oikawa H (2006). Determinant factors of spectrum of missense variants in mucopolysaccharidosis IVA gene. Mol Genet Metab.

[4] Montano AM, Sukegawa K, Kato Z, Carrozzo R, Di Natale P, Christensen E (2007). Effect of ‘attenuated’ mutations in mucopolysaccharidosis IVA on molecular phenotypes of *N*-acetylgalactosamine-6-sulfate sulfatase. J Inherit Metab Dis.

[5] Ben Turkia H, Tebib N, Azzouz H, Abdelmoula MS, Ben Chehida A, Chemli J, Monastiri K, Chaabouni M, Sanhagi H, Zouari B, Kaabachi N, Ben Dridi MF (2009). Incidence of mucopolysaccharidoses in Tunisia. Tunis Med.

[6] Stone JE (1998). Urine analysis in the diagnosis of mucopolysaccharide disorders. Ann Clin Biochem.

[7] van Diggelen OP, Zhao H, Kleijer WJ, Janse HC, Poorthuis BJ, van Pelt J (1990). A fluorimetric enzyme assay for the diagnosis of Morquio disease type A (MPS IV A). Clin Chim Acta.

[8] Green MR, Sambrook J (2012). Molecular cloning: a laboratory manual.

[9] Iwata H, Tomatsu S, Fukuda S, Uchiyama A, Rezvi GM, Ogawa T (1995). Mucopolysaccharidosis IVA: polymorphic haplotypes and informative RFLPs in the Japanese population. Hum Genet.

[10] Rezvi GM, Tomatsu S, Fukuda S, Yamagishi A, Cooper A, Wraith JE (1996). Mucopolysaccharidosis IVA: a comparative study of polymorphic DNA haplotypes in the Caucasian and Japanese populations. J Inherit Metab Dis.

[11] Tomatsu S, Fukuda S, Uchiyama A, Hori T, Nakashima Y, Sukegawa K (1995). Polymerase chain reaction detection of two novel human N-acetylgalactosamine-6-sulfate sulfatase gene polymorphisms by single-strand conformation polymorphism analysis or by StyI and StuI cleavages. Hum Genet.

[12] Tomatsu S, Fukuda S, Iwata H, Ogawa T, Sukegawa K, Orii T (1994). XhoI and SphI RFLPs in the GALNS gene. Hum Mol Genet.

[13] Bunge S, Kleijer WJ, Tylki-Szymanska A, Steglich C, Beck M, Tomatsu S (1997). Identification of 31 novel mutations in the *N*-acetylgalactosamine-6-sulfatase gene reveals excessive allelic heterogeneity among patients with Morquio A syndrome. Hum Mutat.

[14] Ben M’rad L and Chalbi N. Le choix matrimonial en Tunisie est-il transmissible? Antropo. 2004;7:31-37.

[15] Laradi S, Tukel T, Khediri S, Shabbeer J, Erazo M, Chkioua L (2006). Mucopolysaccharidosis type IV: *N*-acetylgalactosamine-6-sulfatase mutations in Tunisian patients. Mol Genet Metab.

[16] Morrone A, Tylee KL, Al-Sayed M, Brusius-Facchin AC, Caciotti A, Church HJ (2014). Molecular testing of 163 patients with Morquio A (Mucopolysaccharidosis IVA) identifies 39 novel GALNS mutations. Mol Genet Metab.

[17] Khedhiri S, Chkioua L, Elcioglu N, Laradi S, Miled A (2014). Mutations and polymorphisms in *N*-acetylgalactosamine-6-sulfate sulfatase gene in Turkish Morquio A patients. Pathol Biol.

